# Development of questionnaire on awareness and action towards symptoms and risk factors of heart attack and stroke among a Malaysian population

**DOI:** 10.1186/s12889-019-7596-1

**Published:** 2019-10-16

**Authors:** Abdullah Abdulmajid Abdo Ahmed, Abdulkareem Mohammed AL-Shami, Shazia Jamshed, Abdul Rahman Fata Nahas

**Affiliations:** Department of Pharmacy Practice, Kulliyyah of Pharmacy, International Islamic University Malaysia, 25200 Kuantan, Pahang Malaysia

**Keywords:** Reliability, Validity, Questionnaire development, Awareness, Heart attack, Stroke, Symptoms, Risk factors, Kuantan, Malaysia

## Abstract

**Background:**

The awareness of symptoms and action towards heart attack and stroke is important to reduce the morbidity and mortality in both developed and developing countries. The aim of this study was to develop a questionnaire on awareness and action towards symptoms and risk factors of heart attack and stroke among lay public in Malaysia. The questionnaire was developed in both English and Bahasa Melayu.

**Methods:**

Primarily the questions were generated in English. Face and content validity were performed by five experts in Pharmacy Practice and Medicine. A translation as per guidelines into Malay language was performed; followed by face-to-face interview of 96 lay public in Kuantan, Pahang, Malaysia. For internal consistency, reliability was assessed utilizing Cronbach’s alpha.

**Results:**

The mean ± SD of the awareness and action towards heart attack symptoms and risk factors was 65.52 ± 6.3, with a good internal consistency (Cronbach’s alpha = 0.75), whereas the mean of the awareness and action towards stroke symptoms and risk factors was 61.93 ± 7.11, with an accepted internal consistency (Cronbach’s alpha = 0.86).

**Conclusion:**

The current validation research showed that the developed questionnaire is valid and reliable for assessing the awareness and action towards symptoms and risk factors of heart attack and stroke among lay public in Malaysia.

## Background

Cardiovascular diseases (CVDs) have been documented as the leading cause of mortality worldwide. The World Health Organization (WHO) reported that about 17.9 million individuals passed away due to CVDs in 2016, which accounted for 31% of all deaths in the world. Out of this number, 85% (15.2 million) were attributed to heart attack and stroke [1]. In fact, stroke is a major problem in both developing and developed countries; however, it is documented that mortality rate increases since the time elapsed from the first onset of stroke symptoms, making early hospitalization a crucial factor to decrease morbidity and mortality. Additionally, better control of risk factors associated with stroke provides better management and prevention. Thus, it is imperative to improve public awareness regarding stroke and its risk factors such as hypertension, diabetes, smoking, stress and physical inactivity [2].

On the other hand, coronary heart disease (CHD), has contributed significantly to mortality cases [3]. With respect to Malaysia, CHD was reported by WHO to account for 23.1% (29363) deaths of all mortality cases in 2014 [4]. With regard to the statistics on the causes of mortality in Malaysia in 2017, ischemic heart disease accounted for 13.9% of all deaths [5]. Myocardial Infarction (MI) is a time-dependent illness, in which the patients’ admission to the hospital at early time yields a significant positive outcome, while pre-hospital delay affects negatively in all patients with respect to their quality of life. For instance, primary percutaneous coronary intervention rely on the time the symptoms appear and their management; therefore, early symptoms recognition and prompt care are important [6]. In fact, post MI symptoms onset, each half an hour delay in reperfusion results in increase in mortality rate by 1.5% [7]. In contrast, the mortality rate has been shown to improve by 23% when reperfusion occurs within three hours and by 50% within one hour, following MI symptoms onset [8].

In fact, there are several modifiable risk factors that lead to CHD, such as unhealthy foods/drinks (e.g., fast foods, trans fats, fizzy drinks), obesity, lack of physical activity, smoking, alcohol consumption, hypertension, diabetes, and high cholesterol. On the other hand, awareness towards CVDs, i.e., heart attack and stroke, and its controllable risk factors is imperative, as it might lead to improvement in individual’s lifestyle and motivation to seek medical assistance at emergency department, which if done at early stage can lead to remarkable reduction of morbidity and mortality [9]. This highlights the importance of exploring individual’s knowledge regarding CVDs; however, few studies assessed CVDs risk factors knowledge among general public [10–12]. Furthermore, and focusing in Malaysia, there is a paucity of research which focus to explore the awareness towards symptoms and risk factors of heart attack and stroke in lay public of Malaysia. However, prior to run main research, a development of questionnaire in English and Bahasa Melayu (BM) languages was performed.

The main objective of this study is to develop a questionnaire to assess the awareness and action towards symptoms and risk factors of heart attack and stroke among the lay public. The current research also focused in translating the developed questionnaire into BM language, and to examine its validity and reliability (internal consistency), as well as to assess the awareness among the respondents in this pilot study.

## Methods

### Design and participants

This was a cross-sectional pilot study that was performed among lay public in Kuantan, Pahang, Malaysia. As suggested by Rattray and Jones [13], a sample size of fewer than 100 subjects is adequate for questionnaire design and development. Hence, a total of 96 individuals was recruited through convenience sampling by visiting crowded areas such as markets and shopping malls, but all healthcare facilities were excluded. Those who were 18–64 years old were included while any healthcare professionals or academicians related to any healthcare discipline were excluded. Participants were interviewed by two trained personnel, who speak both BM and English. The study objectives were fully explained to all participants who provided a written informed consent. All participants were assured that their confidentiality to be maintained, and that information collected will only be used for research purposes. The study was approved by the International Islamic University Malaysia Research Ethics committee (IREC 2018–132, dated March 12, 2018).

### Questionnaire development

The questionnaire was first developed in English language, which was adapted from several studies that had been conducted among different races worldwide (Australia, Greece, Iran, Japan, and Korea; [6, 14–18]). We also used questionnaires from two studies that were conducted among all races in Penang, Malaysia [9, 11]. The questions then strengthened to address the study objectives. The initial questionnaire consisted of 18 questions: 11 on awareness and action towards symptoms and risk factors of heart attack, and 7 on awareness and action towards symptoms and risk factors of stroke. However, the final questionnaire comprised of 14 questions; eight questions on awareness and action towards symptoms and risk factors of heart attack and six questions on awareness and action towards symptoms and risk factors of stroke. Demographic characteristics were also addressed.

The final draft of the questionnaire was established after sending it to five professionals in this field, who evaluated it for face and content validity. Necessary amendments were done after the inclusion of experts’ opinions.

### Translation

In concordance with the guidelines recommended by Guillemin et al. [19] and Beaton et al. [20], the BM-version of the questionnaire was generated. Firstly, two qualified independent linguistic translators, whose mother tongue is BM and proficient in English, translated the questions from English to BM. To ensure the quality of the translation, only one of the translators knew the purpose of the study. Following that, a Malaysian expert who works as a researcher revised the two primary versions and compared them with the original English version. As a result, the first version of the Malay questionnaire was made.

In the second stage, back translation was performed from BM to English by another two Malaysian translators, who were proficient in both English and BM and did not know the purpose of the study. The back-translated English questionnaire was compared with the original English questionnaire and, as a result, the second version of the English translated questionnaire was made.

The last stage included the distribution of BM questionnaire to 10 lay public respondents, who answered and commented on the questionnaire.

The researchers further discussed all questionnaire versions**,** while also considered respondents’ comments. Necessary amendments were made, yielding the final BM version of the questionnaire for the reliability and validity study.

### Statistical analysis

The data were analyzed after they were collected using Statistical Package for Social Sciences (SPSS version 25), and descriptive and inferential statistics applied. Descriptive analyses were performed to describe the variables. A reliability test was performed to ascertain Cronbach’s alpha coefficient (internal consistency and corrected item-total correlation). Mann-Whitney U test and Kruskal-Wallis test were also carried out to address the relationship between variables and awareness of all five-heart attack and stroke symptoms.

## Results

### Data description

A total of 96 participants were included in the final analysis. Majority of them were males (*n* = 52, 54.2%). Thirty-three participants (34.4%) aged 18 to 25 years old, 25% (*n* = 24) aged 26 to 35 years old, 18.8% (*n* = 18) aged 36 to 45 years old, 9.4% (*n* = 9) aged 46 to 55 years old and 12.5% (*n* = 12) aged 56 to 64 years old. Most of participants were single (*n* = 51, 53.1%), while 51% participants (*n* = 49) reported high school education. Most of them were employed (*n* = 59, 61.5%), Malay (*n* = 74, 77.1%) and with a monthly income of less than US$ 500 (*n* = 66, 68.8%). Half of the participants reported to be healthy (*n* = 48, 50%). Of the participants, 13.5% had hypertension (*n* = 13), 11.5% had diabetes (*n* = 11), 15.6% had dyslipidemia (*n* = 15), 5.2% had heart disease (*n* = 5) and only four had stroke. Details of participants’ sociodemographic characteristics are shown in Table 1.

**Table 1:** Socio-demographic characteristics of study participants (*N* = 96)

Variables	F	%
Gender		
Male	52	54.2
Female	44	45.8
Age (years)		
18–25	33	34.4
26–35	24	25
36–45	18	18.8
46–55	9	9.4
56–64	12	12.5
Marital Status		
Single	51	53.1
Married	45	46.9
Divorced	0	0.0
Widow	0	0.0
Education		
Lower school	7	7.3
Seconder school	49	51
Undergraduate	12	12.5
Diploma	18	18.8
Master	7	7.3
Others	3	3.1
Employment Status		
House wife	2	2.1
Employed	59	61.5
self-employed	13	13.5
Unemployed	3	3.1
Student	13	13.5
Retired	6	6.3
Race		
Malay	74	77.1
Chinese	16	16.7
Indians	3	3.1
Others	3	3.1
Monthly income		
< 500 USD	66	68.8
500-less1000 USD	17	17.7
1000–1500 USD	13	13.5
> 1500 USD	0	0.0
Hypertension	13	13.5
Diabetes	11	11.5
Dyslipidemia	15	15.6
Heart diseases	5	5.2
Stroke	4	4.2
Other diseases	2	2.1

### Reliability

#### Internal consistency reliability

Mean of the awareness and action towards symptoms and risk factors of heart attack was 65.52 ± 6.36, and for stroke was 61.93 ± 7.11. Additionally, Cronbach’s alpha of internal consistency was 0.79 and 0.83, respectively, which reveal good reliability of both tools. The internal consistency was determined by eight questions on awareness and action towards symptoms and risk factors of heart attack and six questions on awareness and action towards symptoms and risk factors of stroke. The item-total correlations between questions of both tools are demonstrated in Tables 2 and 3.

**Table 2:** Reliability tests of 8 items of awareness and action toward heart attack symptoms and risk factors

Questions	Corrected Item-Total Correlation	Cronbach’s Alpha if Item Deleted
Q1-Have you ever heard about heart attack?	0.372	.785
Q2-Do you know anyone who had heart attack before?	0.274	.786
Q3- Have you ever received any information related to heart attack? (e.g. Heart attack-related public service announcements, promotional materials, educational leaflets or attended any seminar on heart attack) A- Yes or NO If yes, please specify: What the source of information you received?	0 .497	.779
3b-have you ever received any information related to heart attack by TV?	.275	.786
3c-have you ever received any information related to heart attack by internet?	.238	.788
3d-have you ever received any information related to heart attack by Books?	.145	.790
3e-have you ever received any information related to heart attack by media?	.302	.785
3f-have you ever received any information related to heart attack by promotional leaflets?	.138	.790
3 g-have you ever received any information related to heart attack by Health care professionals?	.188	.789
3 h-have you ever received any information related to heart attack by seminars?	.225	.788
3i-have you ever received any information related to heart attack by others?	.096	.791
Q4- How do you identify the symptoms of heart attack? If you think the following symptoms are related to the symptoms of heart attack, please answer ‘Yes’; if you do not think so, answer ‘No’; if you are unsure, answer ‘I do not know’.		
4a- Do you think sudden pain or discomfort in jaw, neck, or back?	.359	.782
4b- Do you think weakness or dizziness?	.438	.778
4c-Do you think sudden pain or discomfort able in the chest?	.345	.783
4d-Do you think sudden disturbance of vision in one or both eyes?	.251	.789
4e- Do you think sudden pain or discomfort in arms or shoulders?	.353	.783
4f- Do you think sudden shortness of breath?	.299	.785
Q5-sudden heart attack requires a prompt treatment?	.318	.785
Q6-If someone shows signs and symptoms of heart attack, what do you think should do first?		
6a-Take them to hospital.	−.137	.801
6b- Call his/her doctors	−.217	.794
6c- Call an ambulance.	.050	.794
6d- Call police.	.047	.791
6e- Contact their family.	−.148	.793
6f- Other actions?	.119	.791
6 g- Do not know	−.378	.794
Q7 (If you want to call an ambulance do you know the phone number	.274	.786
7a-If yes (Please specify the phone number)	.271	.786
Q8-Have you heard about the risk factors of heart attack?	.363	.784
8a- Do you think smoking are the major factor of heart attack?	.242	.787
8b- Do you think obesity are the major factors of heart attack?	.335	.784
8c- Do you think diabetes are the major factors of heart attack?	.349	.784
8d- Do you think exercise are the major factors of heart attack?	.301	.785
8e- Do you think unhealthy diet are the major factors of heart attack?	.358	.783
8f- Do you think stress are the major factors of heart attack?	.455	.780
8 g- Do you think high blood pressure are the major factors of heart attack?	.504	.777
8 h- Do you think alcohol is the major factors of heart attack?	.366	.783
8i- Do you think genetic is the major factors of heart attack?	.249	.787
8j- Do you think atrial fibrillation is the major fact of heart attack?	.311	.785
8 k- Do you think high cholesterol is the major factor of heart attack?	.371	.783
8 l- Do you think lack of exercise is the major factors of heart attack?	.316	.785
8 m- Do you think heart disease is the major factor of heart attack?	.193	.789

**Table 3:** Reliability test of 6 questions of awareness and action towards stroke signs, symptoms and risk factors

Questions	Corrected Item-Total Correlation	Cronbach’s Alpha if Item Deleted
Q1-Have you heard about term of stroke?	.418	.857
Q2-Do you know anyone who had stroke?	.522	.852
Q3- How do you identify the warning signs and symptoms of stroke? If you think the following signs and symptoms are related to the symptoms and signs of stroke, Pleas answer ‘Yes’; if you do not think so, answer ‘No’; if you are unsure, answer ‘I do not know’.		
3a-Do you think sudden confusion, trouble speaking or understanding speech is a stroke symptom?	.440	.856
3b-Do you think sudden nosebleed is a stroke symptom?	.516	.852
3c-Do you think sudden numbness or weakness of face, arm or leg is a symptom of stroke?	.515	.852
3d-Do you think sudden trouble seeing in one or both eyes is a stroke symptom?	.494	.854
3e-Do you think sudden vomiting is a stroke symptom? (trap question)	.451	.854
3f- Do you think sudden trouble walking, dizziness, loss of balance or coordination are stroke symptoms?	.574	.850
3 g-Do you think sudden severe headache with no known cause is a stroke symptom?	.553	.851
3 h-Do you think high temperature is a symptom of stroke?	.549	.851
Q4- How do you identify the risk factors of stroke? (Select more than one option if necessary)		
4a-Do you think Smoking is the risk factor of stroke?	.433	.855
4b-Do you think Cough is the risk factor of stroke? (trap question)	.152	.861
4c- Do you think Lack of exercise is the risk factor of stroke?	.421	.855
4d- Do you think High blood pressure is the risk factor of stroke?	.437	.855
4e- Do you think Heart disease is the risk factor of stroke?	.541	.852
4f- Do you think Family history is the risk factor of stroke?	.399	.856
4 g- Do you think High level of cholesterol is the risk factor of stroke?	.523	.853
4 h- Do you think Obesity or Overweight is the risk factor of stroke?	.561	.852
4i- Do you think Diabetes is the risk factor of stroke?	.344	.857
4j- Do you think Unhealthy diet is the risk factor of stroke?	.489	.854
4 k- Do you think Stress is the risk factor of stroke?	.436	.855
4 l- Do you think an Alcohol is stroke risk factors?	.481	.854
4 m-Do you think Atrial fibrillation is stroke risk factors?	.477	.854
Q5-Do you think Stroke requires a prompt treatment?	.189	.860
Q6- If someone shows signs and symptoms of stroke, what do you think you should do first? (Please choose just one)		
6a-Give them Aspirin	.000	.862
6b- Contact him/her family	−.025	.863
6c- take them to the hospital, or clink	−.235	.871
6d- Call an ambulance) (this is the appropriate action	.047	.864
6e- call health care provider	.011	.862

#### Test-retest reliability

Investigation of the pre-test and post-test of the reliability with a period of 2 weeks for 10 participants displayed satisfactory reliability and stability, with a Spearman’s rank correlation coefficient value equals to 0.775 (*p* = 0.008) for awareness and action towards symptoms and risk factors of heart attack and 0.954 (*p* = 0.00) for awareness and action towards symptoms and risk factors of stroke.

### Validity

#### Awareness of heart attack groups validity

Married participants showed higher awareness on all five symptoms of heart attack compared to single participants (*P* = 0.033). Furthermore, Kruskal-Wallis test was performed to examine the differences on awareness of all five heart attack symptoms according to education, monthly income, race, and employed status. We found a significant difference between those who have high and low monthly income, respondents who have higher income (1000-1500US) illustrated better awareness on five HAS than low income (Chi-square = 6.277, *P* = 0.043, df = 2). However, there was no significant differences between education, race, age and employment status with awareness of five heart attack symptoms (Chi-square = 8.573, *p* = 0.073, df = 4), (Chi-square = 3.303, *p* = 0.347, df = 4), (Chi-square = 2.760, *p* = 0.599, df = 4) and (Chi-square = 7.461, *p* = 189, df = 5), respectively. Moreover, those who had risk factors of CVDs such as diabetes and dyslipidemia showed greater awareness of the five heart attack symptoms (HAS) than those with no CVDs risk factors (*P* = 0.007, 0.002 respectively) as in Table 4.

**Table 4:** Correlation between awareness of all five HAS and participants demographic

Parameters	N	Awareness of all five HAS Mean Rank	Awareness of all five HAS Median	*P*-value
Gender				
Male	52	48.69	4	0.87
Female	44	48.27	3	
Age				
18–25	33	46.45	1	0.60
26–35	24	49.00	2	
36–45	18	47.67	1	
46–55	9	50.33	1	
56–64	12	53.00	2	
Marital status				
Single	51	45.94	1	**0.033**
Married	45	51.40	6	
Race				
Malay	74	48.24	5	0.35
Chinese	16	48.00	1	
Indians	3	45.00	0	
Others	3	61.00	1	
Education				
Lower school	7	50.14	1	0.07
High school	49	44.45	1	
Undergraduate	12	51.25	2	
Diploma	18	46.08	1	
Master	7	56.79	1	
PHD	–	–	–	
Employment				
House wife	2	45.00	0	0.194
Employed	59	48.25	4	
Self-employed	13	45.00	0	
Unemployed	3	45.00	0	
Students	13	48.69	1	
Retired	6	61.00	2	
Monthly income				
Less than 500 US	66	47.91	4	**0.043**
500–1000 US	17	45.00	**0**	
1000–1500 US	13	56.08	3	
Know someone have past heart attack				
Yes	89	51.72	6	**0.009**
No	7	45.00	1	
Diabetes				
Yes	11	58.09	**3**	**0.007**
No	85	47.26	4	
Dyslipidemia				
Yes	15	57.80	4	**0.002**
No	81	46.78	3	

*HAS* heart attack symptoms

Values in bold are statistically significant

#### Awareness of all five heart attack symptoms and appropriate action validity

The Kruskal- Wallis test demonstrated significant differences in social demographic characteristic between awareness of all five HAS and its appropriate action (HAS-A). Respondents who have high education and high monthly income represented good awareness on HAS-A than those with low education and income (Chi-square = 17.801, *P* = 0.001, df = 4) and (Chi-square = 5.050, *P* = 0.08, df = 2), respectively. Mann-Whitney test also showed that those who have diabetes or dyslipidemia had better awareness of all five HAS and its appropriate action (U = 393, *p* = 0.014) and (U = 493, *p* = 0.001), respectively, as shown in Table 5. Likewise, Kruskal-Wallis test displayed significant differences of employment status, in which retired and students showed the highest awareness of all five HAS-A (Chi-square = 14.873, *p* = 0.011, df = 5).

**Table 5:** Differences of HAS-A awareness by social characteristics

Parameters	Number of respondents	Awareness of all five HAS and action (Mean Rank)	Awareness of all five HAS and action (Median)	*P*-value
Education				
Lower school	7	45.00	**0**	**0.001** ^*^
High school	49	45.00	0	
Undergraduate	12	52.00	2	
Diploma	18	45.00	0	
Master	7	58.29	2	
PHD	–	–	–	
Employments				
House wife	2	46.50	**0**	**0.01** ^*^
Employed	59	47.31	1	
Self-employed	13	46.50	0	
Unemployed	3	46.50	0	
Students	13	50.19	1	
Retired	6	62.50	2	
Diabetic				
Yes	11	55.23	**2**	**0.014** ^**^
No	85	47.63	2	
Dyslipidemia				
Yes	15	56.10	**3**	**0.001** ^**^
No	81	47.09	1	

*Kruskal-Wallis test, **Mann- Whitney U test

*HAS* heart attack symptoms, *HAS-A* HAS and appropriate action

Values in bold are statistically significant

Further analysis showed that Malays and Chinese had more awareness of all five heart attack symptoms than Indians; however, there was no statistically significant difference (*P* = 0.352), in addition, Chinese reported more knowledge of all five HAS and action towards HAS than Malays and Indians, with no statistically significant difference (*P* = 0.933; Table 6).

**Table 6:** Differences in awareness and knowledge towards HAS and action among different races

Awareness of HAS and action towards HAS		
Race	N	Mean Rank
Malay	74	47.14
Chinese	16	46.91
Indians	3	44.05
Others	3	61.00
Knowledge of HAS and action towards HAS		
Race	N	Mean Rank
Malay	74	48.89
Chinese	16	49.91
Indians	3	46.00
Others	3	46.00

*Kruskal-Wallis Test *p* < 0.05

Regarding stroke awareness among different races, although no significant difference yielded (*P* = 0.49), Chinese showed the highest awareness towards all five stroke symptoms and action towards stroke, followed by Malays and Indians. (Table 7).

**Table 7:** Differences in awareness towards stroke symptoms and action among different races

Race	N	Mean Rank
Malay	74	46.27
Chinese	16	51.22
Indians	3	42.50
Others	3	

*Kruskal-Wallis Test

#### Awareness of stroke symptom validity

Mann-Whitney U test revealed significant differences between participants who have diabetes, previous heart diseases, dyslipidemia, and past stroke with awareness of all five stroke symptoms (U = 328, *p* = 0.039), (U = 103, *p* = 0.008), (U = 426, *p* = 0.019) and (U = 94, *p* = 0.034), respectively. Those who have risk factors of stroke reported better awareness on all five stroke symptoms than others without these condition as Table 8 shows. However, Kurskal-Wallis Test was done to determine the differences on awareness of all five stroke symptoms with social demographics. There was no significant differences between those with high education and low education (Chi-square = 2.200, *p* = 0.699, df = 4), or those with high monthly income and low monthly income (Chi-square = 4.356, *p* = 0.113, df = 2).

**Table 8:** Relationship between awareness of all five stroke symptoms and participants with risk factors of stroke

Parameters	Number of participants	Mean rank	Median	*P*-value
Diabetes				
Yes	11	61.18	6	**0.039** ^*^
No	85	46.86	21	
Heart diseases				
Yes	5	73.40	4	**0.008** ^*^
No	91	47.13	23	
Dyslipidemia				
Yes	15	60.60	8	**0.019** ^*^
No	81	46.26	19	
Past stroke				
Yes	4	71.00	3	**0.034** ^*^
No	92	47.52	24	

* Mann-Whitney U Test

Values in bold are statistically significant

## Discussion

To the best of our knowledge, this study is the first study in Malaysia to develop and validate BM questionnaire to *assess* the awareness and action towards symptoms and risk factors of heart attack and stroke in lay public. It can be considered a reliable instrument for future research to gauge lay public of the awareness and action towards symptoms and risk factors of heart attack and stroke.

Several questionnaires had been developed for measuring awareness and action towards symptoms and risk factors of heart attack and stroke in different countries. Using such tools can provide interesting findings, yet other factors should be considered that might affect respondents’ responses, such as education, monthly income, age or residency [14, 21–29].

Following the development of the questionnaire in English, we followed the recommended guidelines of Guillemin et al. [19] and Beaton et al. [20] to translate the questionnaire to BM, which is the official language of Malaysia.

The original questionnaire developed and validated (face and content validity) to become relatively simple and understandable among Malaysian people. We obtained a reliable questionnaire, as an internal consistency coefficient of the final BM version was good for both heart attack and stroke. Nunally and Bernstein [30] recommended a minimum Cronbach’s alpha value of 0.70 for a reliable questionnaire.

The awareness group validity represented that participants who had high level of education, high monthly income and risk factors of heart attack and stroke showed higher awareness on all five-heart attack and stroke symptoms than others without these conditions. The significant differences between the total awareness of all five symptoms of both heart attack and stroke of those who had high level of education and income as well as those who have risk factors of HAS and stroke symptoms revealed that the questionnaire had a good construct validity.

The result of this study showed that the developed translated questionnaire measures the awareness of symptoms of heart attack and stroke among general Malaysian people. Study findings also revealed that how education, monthly income and risk factors of both HA and stroke can impact the awareness of heart attack and stroke symptoms, which was similar to studies conducted in the US [28] and Korea [31]. These studies reported that respondents who had high education, high monthly income and risk factors of heart attack have good awareness of heart attack symptoms.

Current study reported that participants who aged 35 to 64 had better awareness of HA symptoms; however, this difference was not significant, which probably due to the fact that older participants had more experience in life and more educated than younger participants; consistent with other studies in Korea [31] and the US [24]. Moreover, there was a significant association between awareness of all five-heart attack and appropriate action with employment status. Students and retired showed more awareness of all five heart attack symptoms and appropriate action than housewives, employed/unemployed individuals that was similar with a study in Singapore [3]. Additionally, the findings of the current study reported no significant differences with gender, age, and race.

With regards to stroke domain, significant differences between respondents who have risk factors of stroke and awareness of all five stroke symptoms were noted; presumably because participants who have risk factors of stroke have more knowledge of symptoms and risk factors, or either they received extensive information from their healthcare professionals. No significant differences between awareness of all five stroke symptoms and gender, education, employment status and monthly income reported.

### Strengths and limitations

This is the first study that comprehensively explored lay public awareness and action towards heart attack and stroke in Malaysia. Being reliable and valid, the questionnaire developed in this study can also be successfully used in future research. A limitation of our study is the moderately small sample size; however, according to previous research, a sample of fewer than 100 subjects is adequate for a pilot study. A second limitation is the convenience sampling that could result in selection bias introduced by the interviewers.

## Conclusion

The current pilot study shows that the developed questionnaire is a reliable and valid measure of all aspects of participants’ awareness of symptoms and action towards heart attack and stroke. It can be also useful in studying the relationship between respondents’ sociodemographic and their awareness and action towards symptoms and risk factors of heart attack and stroke.

## Data Availability

The data related to this study is a part of larger data of a master’s degree project. Data is available only upon request. The Bahasa Melayu (Malay language) questionnaire is also available from the authors upon request.

## Abbreviations

BM: Bashas Melayu
CHD: Coronary heart disease
CVDs: Cardiovascular diseases
HA: Heart attack
HAS: Heart attack symptoms
HAS-A: Heart attack symptoms and appropriate action
WHO: World Health Organization

## References

[1] WHO (2018). Cardiovascular diseases (CVDs).

[2] Das K, Mondal GP, Dutta AK, Mukherjee B, Mukherjee BB (2007). Awareness of warning symptoms and risk factors of stroke in the general population and in survivors stroke. J Clin Neurosci.

[3] Quah JLJ, Yap S, Cheah SO, Ng YY, Goh ES, Doctor N, et al. Knowledge of signs and symptoms of heart attack and stroke among Singapore residents. Biomed Res Int. 2014;2014:8. Article ID 572425. https://www.hindawi.com/journals/bmri/2014/572425/cta/. 10.1155/2014/572425 PMC4000924 24812623

[4] Abdullah W, Yusoff Y, Basir N, Yusuf M (2017). Mortality rates due to coronary heart disease by specific sex and age groups among Malaysians. Proceedings of the world congress on engineering and computer science.

[5] Malaysia Dos (2018). Statistics on causes of death, Malaysia.

[6] Intas G, Tsolakoglou J, Stergiannis P, Chalari E, Eleni C, Fildissis G (2015). Do greek citizens have minimum knowledge about heart attack? A survey. J Health Sci.

[7] McNamara RL, Wang Y, Herrin J, Curtis JP, Bradley EH, Magid DJ (2006). Effect of door-to-balloon time on mortality in patients with ST-segment elevation myocardial infarction. J Am Coll Cardiol.

[8] Simoons ML, Serruys PW, van den Brand M, Res J, Verheugt FW, Krauss XH (1986). Early thrombolysis in acute myocardial infarction: limitation of infarct size and improved survival. J Am Coll Cardiol.

[9] Awad A, Al-Nafisi H (2014). Public knowledge of cardiovascular disease and its risk factors in Kuwait: a cross-sectional survey. BMC Public Health.

[10] Muhamad R, Yahya R, Yusoff HM (2012). Knowledge, attitude and practice on cardiovascular disease among women in north-Eastcoast Malaysia. Int J Collaborative Res Int Med Public Health.

[11] Amin AM, Hamza M, Azmi S (2014). Factors associated with the general public knowledge and awareness of cardiovascular diseases and its risk factors in Penang–Malaysia. ISOR Journal of Pharm.

[12] Ibrahim MM, Rahman NAA, Rahman NIA, Haque M (2016). Knowledge, attitude and practice of Malaysian public university students on risk factors for cardiovascular diseases. J Appl Pharm Sci.

[13] Rattray J, Jones M (2007). Essential elements of questionnaire design and development. J Clin Nurs.

[14] Kim H-S, Lee H, Kim K, Park H-K, Park K-S, Kang GW, et al. The general public’s awareness of early symptoms of and emergency responses to acute myocardial infarction and related factors in South Korea: a national public telephone survey. J Epidemiol. 2016;26(5):233-41. 10.2188/jea.JE20150074 PMC4848321 26853101

[15] Miyamatsu N, Okamura T, Nakayama H, Toyoda K, Suzuki K, Toyota A (2013). Public awareness of early symptoms of stroke and information sources about stroke among the general Japanese population: the Acquisition of Stroke Knowledge Study. Cerebrovasc Dis.

[16] Sug Yoon S, Heller RF, Levi C, Wiggers J, Fitzgerald PE (2001). Knowledge of stroke risk factors, warning symptoms, and treatment among an Australian urban population. Stroke.

[17] Hosseininezhad M, Ebrahimi H, Seyedsaadat SM, Bakhshayesh B, Asadi M, Ghayeghran AR (2017). Awareness toward stroke in a population-based sample of Iranian adults. Iran J Neurol.

[18] Park M, Kim K, Lee JH, Kang C, Jo YH, Kim DH (2014). Awareness and knowledge of sepsis in the general Korean population: comparison with the awareness and knowledge of acute myocardial infarction and stroke. Clin Exp Emerg Med.

[19] Guillemin F, Bombardier C, Beaton D (1993). Cross-cultural adaptation of health-related quality of life measures: literature review and proposed guidelines. J Clin Epidemiol.

[20] Beaton DE, Bombardier C, Guillemin F, Ferraz MB (2000). Guidelines for the process of cross-cultural adaptation of self-report measures. Spine (Phila Pa 1976).

[21] Nansseu JR, Atangana CP, Petnga S-JN, Kamtchum-Tatuene J, Noubiap JJ (2017). Assessment of the general public's knowledge of stroke: a cross-sectional study in Yaoundé, Cameroon. J Neurol Sci.

[22] Komolafe MA, Obembe AO, Olaogun MO, Adebiyi AM, Ugalahi T, Dada O (2015). Awareness of stroke risk factors and warning signs in Nigerian adolescents compared with adults. J Stroke Cerebrovasc Dis.

[23] Duque AS, Fernandes L, Correia AF, Calvinho I, Cardoso G, Pinto M, et al. Awareness of stroke risk factors and warning signs and attitude to acute stroke. Int Arch Med. 2015;8. http://imed.pub/ojs/index.php/iam/article/view/1236.

[24] Greenlund KJ, Keenan NL, Giles WH, Zheng ZJ, Neff LJ, Croft JB (2004). Public recognition of major signs and symptoms of heart attack: seventeen states and the US Virgin Islands, 2001. Am Heart J.

[25] Kim EM, Hwang SY, Kim AL (2011). Knowledge of stroke and heart attack symptoms and risk factors among rural elderly people: a questionnaire survey. Korean Circ J.

[26] Khan MS, Jafary FH, Jafar TH, Faruqui AM, Rasool SI, Hatcher J (2006). Knowledge of modifiable risk factors of heart disease among patients with acute myocardial infarction in Karachi, Pakistan: a cross sectional study. BMC Cardiovasc Disord.

[27] Du H, Dong C-y, Lin Q-y (2015). Risk factors of acute myocardial infarction in middle-aged and adolescent people (< 45 years) in Yantai. BMC Cardiovasc Disord.

[28] Fang J, Gillespie C, Keenan NL, Greenlund KJ (2011). Awareness of heart attack symptoms among US adults in 2007, and changes in awareness from 2001 to 2007. Futur Cardiol.

[29] Swanoski MT, Lutfiyya MN, Amaro ML, Akers MF, Huot KL (2012). Knowledge of heart attack and stroke symptomology: a cross-sectional comparison of rural and non-rural US adults. BMC Public Health.

[30] Nunally JC, Bernstein I (1978). Psychometric Theory.

[31] Kim H-S, Lee H, Kim K, Park H-K, Park K-S, Kang GW (2016). The general public’s awareness of early symptoms of and emergency responses to acute myocardial infarction and related factors in South Korea: a national public telephone survey. J Epidemiol.

